# School nurses’ experience of communicating growth data and weight development to parents of children 8 and 10 years of age

**DOI:** 10.1186/s12889-022-14941-9

**Published:** 2023-01-04

**Authors:** Caroline Skantze, Gerd Almqvist-Tangen, Staffan Karlsson

**Affiliations:** 1 School of Health and Welfare, Halmstad University, PO Box 823, 30118 Halmstad, Sweden; 2 Department of Paediatrics, University of Gothenburg, 40530 Gothenburg, Sweden; 3 Faculty of Health Sciences, Halmstad University and Kristianstads University, 29188 Kristianstad, Sweden

**Keywords:** School nurses, Parents, Communication, Growth data, Weight development, Children

## Abstract

**Introduction:**

The prevalence of overweight and obesity among children has risen sharply during recent decades. School nurses are key health professionals in interventions targeting the early onset of overweight and obesity during childhood. Understanding how school nurses experience communication with parents concerning their child´s growth and weight development are essential. The aim of the study is to describe school nurses’ experience of communicating growth data and weight development to parents of school children ages 8 and 10 years.

**Method:**

The design of the study is a descriptive, qualitative design with purposive and snowball sampling. Sixteen interviews with school nurses were conducted and analysed with qualitative content analysis.

**Result:**

The analysis resulted in three main categories including subcategories. In *Challenges in the professional role,* the school nurses expressed a lack of knowledge, skills and tools in communication, described a perception of parental responsibility and stated using several different approaches in communicating growth data and weight development to parents. In *Sustainable communication with parents,* the school nurses described the value of creating a dialogue, a supportive approach to the parents, and the building of a relation between them and the parents as essential. In *Barriers in communicating the child´s weight*, the school nurses described the experience of stigma concerning the subject of overweight and obesity, increased concern when they detected underweight as well as an ambivalence towards measuring weight.

**Conclusion:**

The study highlights an educational challenge concerning the need for training, skills, and strategies for communication with parents. A variety of ways in school nurses’ communication with parents were identified in the present study and this shows an inconsistency in how School health services was offered and a need for the development of evidence-based procedures for communicating growth data and weight development to parents.

## Introduction

Childhood overweight and obesity has become a serious worldwide health problem during recent decades. According to the World Health Organisation [1], it is a priority in public health work to prevent and counteract, not just overweight and, obesity in children and adolescents, but also underweight. The prevalence of overweight and obesity among children and adolescents aged 5–19 has risen sharply from 4% in 1975 to over 18% in 2016 [1]. The Organisation for Economic Co-operation and Development (OECD) estimated in 2018 that one in five (19%) 15-year-old children were overweight or obese on average, across 33 EU countries [2].

Children with obesity are at increased risk for obesity as adults and childhood obesity is linked to diseases, such as metabolic syndrome, Type 2 Diabetes Mellitus, hypertension, dyslipidaemia, obstructive sleep apnoea, fatty liver, and colon cancer [3, 4]. In addition, to have an elevated Body Mass Index (BMI) as a child and adolescent has been associated to an increased risk of cardiovascular disease and development of cancer as an adult [5–7]. According to the International Obesity Task Force (IAOTF) the recognised definition of BMI in children, is ISO-BMI 25 when overweight and ISO-BMI 30 when obese [8]. Although BMI may not be an optimal measurement method, currently there is no other way to compare the prevalence of obesity between different countries or within them. Individuals (*n* = 7049) between 3 – 17,9 years in the Swedish child obesity register (BORIS) were compared to a population based-comparison group (*n* = 34 310) and the result indicates an increased risk of higher mortality in early adulthood [9]. Studying BORIS data from 2004 and 2017 (*n* = 21,499) the data show no trend of improvement in the obesity treatment results and no difference between the sexes [10]. Early intervention during the onset of obesity in childhood has the most potential to prevent obesity and other long-term health consequences for high-risk children was indicated in studies from Denmark and Germany [7, 11]. Overall, the accumulated knowledge indicates that overweight and obesity in children increases the risk of morbidity and poor health as adults.

It is essential to understand how school nurses perceive communication with parents about children´s growth and weight development. According to research by Abdin et al. [12] health professionals experience discussing the issue of children´s overweight and obesity with families and children as sensitive. Strategies for communicating are needed, but child weight issues should also be viewed as a shared responsibility between organisations and professionals, rather than the responsibility of a single profession [12]. A Swedish study highlighted that school nurse´s role was transformed towards more health promotion indicated a need for enhanced relational and communication skills but also a structural collegial supervision that could support the school nurses in their professional approach [13]. As shown by Francis et al. [14], school nurses (*n* = 210) claim that they lack communication tools when discussing overweight and obesity issues with parents. The majority (71.4%) found they would benefit from a BMI toolkit in communication with parents. When informing parents of BMI results, the majority (81%) of the school nurses did not offer a follow up, or they only started working with parents if and when the BMI screening showed overweight or obesity [14]. According to a study from Finland the communication between parents and school nurses was perceived as insufficient by parents, as they wished to be informed both orally and in writing by the school nurses concerning the growth of their child [15]. In a study conducted in US the majority (67.5%) of parents to 5—12 year-old children (n = 117) wanted to receive a letter from the school nurse regarding their child’s BMI [16]. Another study from US showed that, regardless of whether the child had overweight or obesity parents wanted the BMI chart of their child to be sent home and expressed that more information on healthy behaviour should be provided by the school nurse [17]. The exchange of information and cooperation between School health services (SHS) and parents regarding their child´s growth is requested by both stakeholders. To our knowledge there are no studies on the condition of communication between school nurses and parents in Sweden. The aim of the study was thus to describe school nurses’ experience of communicating growth data and weight development to parents of school children ages 8 and 10 years.

## Method

### Design

The design of the study was a descriptive, qualitative design.

### Participants

Sixteen school nurses participated in the study. Fourteen nurses were employed by the municipality and, two by independent school companies. Request of participation was sent to all elementary schools in five different municipalities in the south-western region of Sweden. The recruitment of school nurses determined the selection of schools. Inclusion criteria were school nurses that served pupils between 6 to 11 years of age and performed health visits with children aged 8 and 10 years. Exclusion criteria were school nurses with bachelor’s degree and school nurses with less than one year of experience. An approval by the head of SHS in each municipality and at both independent schools was obtained. All the 16 participating school nurses were recruited via e-mail forwarded to them by the head of SHS in each municipality and independent school, with written information and a request for participation in the study. Contact by interested school nurses was made directly to the first author, and the date for the interview was decided over e-mail or telephone. Two school nurses were recruited by other participants, so-called snowball sampling.

### Context

SHS in Sweden is defined by the Education Act [18] as an overall function between medical, psychological, psychosocial and special education efforts. SHS is governed by laws and regulations and monitored nationally by The National Board of Health and Welfare, and the National Agency of Education [19]. The municipality or provider of independent schools organises SHS in a manner that suits local conditions, and the school nurses are either employed by the school organiser or the function is outsourced to an external partner. In each school the principal is responsible for leading and coordinating SHS, and the head of administration for children and education monitors SHS in the municipality. The Convention on the Rights of the Child [20], has been incorporated into Swedish law since 2020, and the best interests of the child shall be a primary consideration. When the patient is a child, the Patient Act [21], states that the child as well as the child’s guardian shall receive any relevant information (ch3, § 3).

SHS is carried out by a school nurse with public health competence and a school physician. The aim of SHS is to increase cooperation, and emphasise health promotion and preventive work, where the goal is to create a positive learning situation for the pupil. In the basic programme, SHS is required to provide an open reception for children in order, to monitor vaccination and offer at least three evenly distributed health visits, commonly at the age of 8, 10 and 12 years old. These health visits include a health conversation assessing general health, growth, sight, hearing, development, and learning. The purpose of the health visits is to as early as possible detect signs of ill-health and growth deviation and take anthropometric measurements such as height, weight, and BMI. In addition, risks, and protective factors such as eating and sleeping habits or physical activity are evaluated according to Guidance by The National Board of Health and Welfare and The National Agency of Education [19].

### Data collection

Data were collected through interviews. A pilot interview was carried out to investigate the relevance of the interview questions and the questions were not changed after the pilot interview. The questions turned out to be well suited. The school nurses were asked to explain their working procedure at a health visit with the child and the experience any ensuing communication with the parents. Further questions concerned the school nurses’ experience of communicating growth deviation to parents, how communication with parents influences healthy weight in children and any suggestions of methods when communicating with parents. The interviews were conducted between June and November 2020 and took place at the elementary school in the school nurse’s work office. Written and oral information about the study, voluntary participation, and confidentiality were given prior to each interview; all interviews were conducted by the first author. The interviews were recorded digitally and transcribed verbatim by the first author. The length of the interviews varied between 12 to 42 min with a median length of 25 min.

### Analysis

The material was analysed following the qualitative content analysis [22]. The transcribed interviews were read through entirely by the first author and discussed within the research group. According to Graneheim and Lundman [22], a manifest content analysis focuses on evident and visible units in the text. This means that units with a central meaning related to the aim were selected in the material. The process of analysis continued with condensation to reach condensed meaning units labelled with a code by the first author. The various codes were compared based on differences and similarities, all authors were involved in discussion of the codes, subcategories and categories until consensus was reached and sorted into six sub-categories and three categories, which constitute the manifested content. (Table 1).

**Table 1:** Examples of the analysis process

**Meaning unit**	**Condensed meaning unit**	**Code**	**Subcategory**	**Category**
Yes, and to motivate parents, i.e., how to reach them and what advice and concrete tips that work. You have a lot of knowledge, but it would be easier	Would be easier if you had concrete advice to motivate parents with	Lack of tools to motivate parents with	Lack of communication skills and tools	Challenges in the professional role
And then at grade two and grade four (children) are too young to talk to about (their) weight, it is the parents you should reason with	The children are too young, it is the parents you should reason with	Reason with the parents not the children	Perception of parental responsibility	
It varies a bit, sometimes I send a note home and ask the parents to call me and sometimes I call them in the afternoon	Varies between calling and sending a note to the parents	Varies in how to make contact with the parents	Various approaches of communication	

### Ethical consideration

The study was carried out according to the guidelines laid down in the Helsinki Declaration and was approved by the Swedish Ethical Review Authority, operating region Uppsala (Dnr 2020–00,567). The informed consent of the participants, including recording was obtained prior to each interview.

## Result

The professional experience of the school nurses varied between 1.5 years to 23 years. They were all registered nurses with varied further education as either as a school nurse, paediatric nurse, district nurse and/or midwife (Table 2).

**Table 2:** Demographic characteristics and work experience of the school nurses (*n* = 16)

Characteristics	Number
**Sex**	
Female	16
Male	0
**Professional licence, supplementary training**	
District nurse	6
District nurse + School nurse	2
District nurse + Paediatric nurse	1
Paediatric nurse	4
Paediatric nurse + Anaesthetic nurse	1
Midwife	1
Midwife + School nurse	1
**Working experience as a school nurse**	
1–5 years	6
6–10 years	3
11–15 years	4
16–20 years	1
21 -25 years	2
**Employment location**	
Municipal school	14
Independent school	2

In the analysis of the interview transcripts concerning their experiences of communicating growth data and weight development to parents of school children aged 8 and 10 years, three categories, including nine subcategories, emerged from the data (Table 3).

**Table 3:** Categories and subcategories

**Categories**	**Subcategories**
Challenges in the professional role	Lack of communication skills and tools Perception of parental responsibility Various approaches in communication
Sustainable communication with parents	The value of creating a dialogue Supportive approach to parents Establishing relationship to parents
Barriers in communicating the child´s weight	Weight stigma Increased concern when underweight Ambivalence towards measuring weight

### Challenges in the professional role

The school nurses described uncertainty concerning their role in health promotive work, and they also experienced a lack of knowledge in communication. They expressed the parental right to knowledge and information concerning their child and the parental responsibility for their child´s health and growth. Consequently, seven different approaches to communicating growth data and weight development to parents emerged in the results.

#### Lack of communication skills and tools

The school nurses described uncertainty concerning their role in health promotive work and the lack of updated guidelines regarding how to make the necessary contact in order to initiate and keep a functioning dialogue with the parent. *“No, we have guidelines and recommendations on how often we should take extra check-ups and so on, however in the contact with the parents something is lacking”* (Interview 2). The school nurses also experienced that the parents need time to absorb and process the information given. They considered it helpful to have a growth chart in front of them and be transparent, using noncritical language when talking to the parents, which could lead to the school nurse and parents cooperating on an individualised approach to the child. *“As a school nurse it would be easier if we had access to concrete tools and phrases to use in order to motivate parents but also a knowledge of which of these interventions that actually functions”* (Interview 14).

#### Perception of parental responsibility

The school nurses experienced that the individual child was too young to discuss growth data with them, thus determining that the communication should be with the parents. *“And then at grade two and grade four (children) are too young to talk to them about (their) weight, it is the parents who you should reason with”* (Interview 15). They described not showing the child the growth chart, experiencing that it was a parental responsibility and could even be considered offensive to talk to the child about weight issues without their parents accompanying them. The school nurses notably highlighted the parental right to information concerning their child’s growth and weight.

They also gave consideration the parental right to oversee decisions regarding their child’s health and that the parents should have the necessary information upon which to base their decisions. *“Yes, I want the parents to participate, it is their children, and it is their responsibility”* (Interview 5). Some thought that this information should not remain within SHS but be communicated directly to the parents.

#### Various approaches to communication

**Fig. 1:**
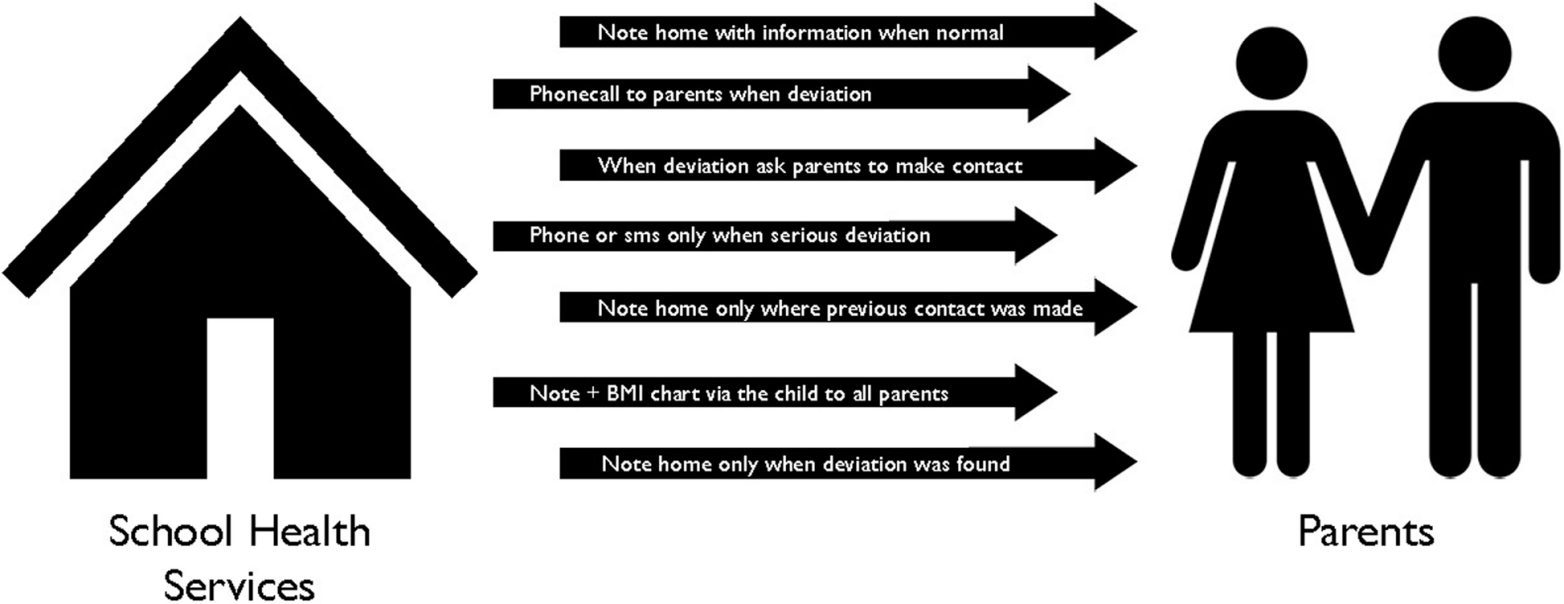
Various approaches of school nurses communicating growth data to parents

The school nurses stated that they use a multitude of approaches when communicating growth data and weight development to parents (Fig. 1). One approach was sending a note to all parents informing of the upcoming health visit, and after the visit was conducted, sending another letter with information showing that the child followed a normal growth chart. If and when the growth chart deviated, they would call the parent by phone until contact was made. *“It varies a bit, sometimes I send a note home and ask the parents to call me and sometimes I call them in the afternoon”* (Interview 9). Another approach was to send a letter home only when the child’s growth deviated from the growth chart, informing parents of the situation, and asking to make contact with them if and when they were worried. The nurses also described making personal contact with parents via telephone when the growth curve deviated in a more serious way or using short messages via mobile phone if the parents were difficult to reach. Some school nurses chose to send a note home only to those parents who had made previous contact and wished to see the growth chart. Still another approach involved only sending a note home to parents when the growth chart deviated according to their professional knowledge. *“And if nothing deviates, I do not communicate anything because it also says in our letter that if something deviates, the school nurse will get in touch one way or another”* (Interview 15). Some also mentioned an approach where they sent a letter home to all parents via the child with the length and growth curve sometimes adding the BMI chart in the letter, as they found this created a continuity in the communication and no parent was missed in the process. According to the school nurses, there had never been a negative response from the parents who received the growth curves. *“No, but I interpret that as something positive because I do not get any negative feedback either. And when I do not hear anything, I interpret it as good”* (Interview 5).

#### Sustainable communication with parents

The school nurses described the dialogue as a work of art that required mastering knowledge, prowess, and experience to master. They experienced the personal meeting, supportive approach, and building of a relation between school nurse and parents as essential.

#### The value of creating a dialogue

The dialogue with the parents was considered vital by the school nurses. The communication between the nurses and parents benefited by an established contact, and the initial personal meeting when the child started preschool was of great value. *“I have come to realise how important it is that you have met the parents at the preschool class meeting”* (Interview 7). Both parents are invited, and both parents are just as important, particularly in divorced families, according to the school nurses. The preschool meeting gave the nurses and parents the possibility to create a structure for how future communication should transpire, thereby building a relationship that the child could benefit from. *“You may even have agreed upon how the communication should take place, because everyone is different”* (Interview 7).

#### Supportive approach to parents

The school nurses expressed that SHS should be regarded as a trusted resource where the parents could find support and help for the issues concerning the growth of their child. They regarded it as essential to examine the whole picture, as well as discussing weight issues with the family, especially when the parents were themselves overweight or obese. However, this required great finger-tip prowess. According to the school nurses, any lifestyle changes should start within the family. “*But how to do it and how to tackle the problem together with parents, this needs great sensitivity and intuition to succeed”* (Interview 9). The school nurses regarded it important that in front of the child it was the parent that made the choices concerning their child and no other authority.

The school nurses were in agreement that the individual child had little influence over their weight or lifestyle changes unless the parents helped them. In other words, the entire family needs to make the necessary changes. Even if the information that was to be given to the parents was painful, they observed and believed that a child’s weight deviation always stood for something. The school nurses stressed the difficulties that arose when no action was taken nor sign of compliance given by the parent and the child was at risk of neglect. In such cases, according to Swedish law, the need to report to Social Services arises. “*Then a report of concern can help many families”* (Interview 14).

#### Establishing a relationship to parents

According to the school nurses, it was necessary to have an open attitude towards the parents and ensure that they were easily accessible by phone. *“You have to somehow get an alliance with the parents. That they feel trusted, and not be critical in any way, but instead to be very clear that you want the best interest for the child and that is why you act the way you do. I think it is very important”* (Interview 16). Most of the school nurses described the cooperation with the parents as vital to positive results and that the work of building relationships should be prioritised. They also expressed that positive encounter were helpful and that the communication between them and the parents should not exclusively concern negative issues.

### Barriers in communicating the child´s weight

The school nurses expressed difficulties, but also concern, in how to communicate the message concerning an overweight child to the parents. They described an experience of stigma regarding the subject of overweight and obesity as well as the increased concern felt when underweight was discovered. An ambivalence concerning the use of BMI among the school nurses emerged in the results in addition the importance of health promotion prior to measured weight.

#### Weight stigma

The issue of at child’s growth, particularly overweight and obesity, is a sensitive topic and the school nurses described it as a difficult conversation to have. They also mentioned an uneasiness concerning how to tackle the subject. *“Somehow it is always more sensitive to talk about it when children are overweight”* (Interview 16). The school nurses experienced the word obesity associated with negative perceptions and connected with guilt. They felt they needed to be careful in the discussions with the parents, although the issue was somewhat easier to address if they first could rule out any medical reason behind the deviating growth chart. “*I usually present in such a way that sometimes an overweight could be due to the fact that there are medical or physical reasons for it”* (Interview 16). A cautious approach was preferred as it was contradictory to the nurse’s profession to do harm. However, they found it necessary to mention the child´s need and desire to have a normal weight. *“You have to start where they are”* (Interview 6).

According to the school nurses, when a child was found to be obese, i.e., with a BMI of 30, a visit to and conversation with the school physician was usually offered to the parents. They also referred to the guideline where a referral to a paediatric outpatient clinic was offered by the school physician. *“If you are overweight, we do not contact the school physician, but if it is obesity, we usually offer an appointment to the school physician and a referral to the paediatric outpatient clinic, and this has happened a couple of times” (*Interview 7).

The school nurses explained that it was up to the parents to manage the contact with the clinic, but if no contact was established, they followed up and made a new proposition to the parent. Their experience was that it sometimes took time to motivate the parent to act upon the referral to a paediatric clinic.

#### Increased concern when underweight

When a child was found to be underweight, the school nurses claimed lack of knowledge and no specified limits for when they should act upon it. In such cases, the school nurses became more concerned when notifying parents, as it gave indication of a severe medical condition *“But underweight is a bit… if you call a parent when a growth chart is stationary, they are very worried and want help”* (Interview 1). It also led to faster action with respect to a visit the school physician or referral to primary health care. The school nurses also described the parents as not always aware of their child’s weight or only able notice it when there was a prominent weight gain or weight loss. Moreover, the school nurses found that parents had difficulties understanding the growth chart and needed an explanation of the length and weight curve, as well as the BMI curve. *“I sometimes notice that parents do not really understand when I say he is a channel over. Nobody understands that and sometimes parents ask, what does he need to weigh at that age?”* (Interview 13).

#### Ambivalence towards measuring weight

The school nurses emphasised the importance of health promotion and a healthy approach to life prior to measuring weight of the child. They experienced that it was more important to discuss health habits and sustainable health behaviour with the parents than stare blindly at the growth chart and discus the child´s weight. *“But also, not to talk so much about weight without health from a health perspective, so it does not become obsessed about weight”* (Interview 15). They explained it as a pursuit surrounding body weight and that they should not problematise children with normal growth. When they explained that the BMI curve was a tool, some parents became annoyed and did not believe in measuring BMI on children. For other parents, the BMI chart was easier to read and understand and could also activate them.

Among the school nurses themselves, a certain disagreement prevailed around the use of BMI. Some conveyed a scepticism of BMI and the fact that BMI was so widely used in SHS, and others considered it a limited measure of weight, although, it was this measure they had in communication with parents. A few school nurses found the BMI curve an important educational tool when communicating with parents. *“I have tried to emphasise that we should maybe work similarly and use the BMI chart, because I think the way we use, it becomes secretive… we have the BMI chart, but we only send home the length and weight chart to the parents”* (Interview 7).

## Discussion

This study seeks to describe school nurses’ experience of communicating children’s growth data and weight development with parents. Three salient categories developed in the results: *Challenges in the professional role, Sustainable communication with parents* and *Barriers in communicating the child´s weight.*

Communication regarding children´s weight development requires a further evolvement of school nurses. In this study, the school nurses described a lack of updated guidelines, knowledge and tools regarding how to make the necessary contact with parents and initiate and maintain a functioning dialogue. A previous study in US, found that school nurses claimed they lacked communication tools when discussing weight issues with parents [14]. Further studies in UK and US confirmed that SHS professionals lack confidence, knowledge, and training regarding how to approach discussing a child’s weight with parents, and the perceived lack of competence in discussing weight-related health posed a barrier for school nurses in their ability to work with and motivate parents [23, 24].

This study shows that school nurses experience uncertainty in their role where health promotive work is concerned. Health professionals in another recent study suggested that child weight issues should be viewed as a shared responsibility between organisations and professionals, rather than the responsibility of one single profession [12]. According to a Guidance by The Swedish National Agency of Education and The National Board of Health and Welfare [19], the goal for SHS is to create as positive a learning situation as possible for the pupil. As SHS is organised according to local conditions, the school nurse can be employed directly at the school or by an external partner. Thus, the platform on which the school nurse operates is not the same between schools, nor is the connection with healthcare always apparent. An official report by the Swedish Government concerning cohesive care for children and adolescences, draws attention to a negative outcome for children as a result of fragmented primary care [25]. This absence of a target image for health promotive work and a clear health perspective regarding overweight and obesity within SHS has consequences for the individual child. The findings indicate an educational challenge concerning the need for training, skills and strategies for school nurses as well as the development of evidence-based procedures and tools for the communication of growth data and weight development to parents.

Parents are the school nurses main target of communication concerning the child´s growth data and weight development. This study showed that the school nurses focused on the collaboration with parents, since the school nurses perceived that they have responsibility for the child’s growth. Further, they experienced the building of a relationship, involving the parents immediately when the child started preschool to create a structure for future communication beneficial for the child. According to Pettersen [26], to build sustainable relationships, a characteristic of ethics of care, and its fundamental principles, constitutes establishing, maintaining, and encouraging caring relationships. To somehow secure an alliance with the parents, where they feel mutual trust and support, was considered essential by the school nurses. This is in accordance with ethics of care, i. e. to strengthen mutual bonds and consider the vulnerability and interest of both parties in the caring relationship [26]. In this study, the school nurses regarded it essential to have a holistic approach when discussing weight issues with the family. The communication between the school nurse and the parents should not only concern negative issues, and they found positive encounters were helpful. This result was confirmed in another study where health care professionals recognised that trust is built up step by step only when a channel of communication is open, and positive language and a gentle approach is crucial [27].

The parental right to information concerning a child’s health and growth was perceived as central by the school nurses in this study. They also recognised that this information should be communicated directly to parents, which is in accordance with previous research in Norway, on how both information and feedback concerning the actual weighing process and weight notification was communicated to parents, where parents did not consider themselves having received sufficient information [28]. The Convention on the Rights of the Child states the child’s right to have the highest attainable standard of health and the need to develop preventive health care and give guidance for parents [20]. The result of this study corresponds with the Parental Code [29], where the parent has the right and obligation to decide in questions regarding their child. The Guidance by The Swedish National Agency of Education and The National Board of Health and Welfare [19] states that a cooperation is recommended between SHS and the parents, but it is unclear what information needs to be communicated and how. The school nurses in this study stated having used a variety of approaches to communicate growth data and weight development to parents. The choice of approach was based partly on the school nurses’ experience and influenced by what they found practically functional and the actual measured weight. This was in line with a previous study of SHS professionals’ experience of addressing childhood obesity, where participants expressed frustration concerning the lack of support for referrals of children identified as overweight. Moreover, although growth charts were used to identify whether a child was overweight, they drew on their “professional judgement” when deciding what action to take [24]. This highlights a problem-solving approach that may be grounded more in functionality than evidence-based practice. Furthermore, the disparate approach of communicating health issues and growth development to parents indicates a discrepancy between the perception regarding the parental right to information and how SHS is offered. To improve SHS, a need for developed national guidelines is indicated, which may correspond to The Swedish National Handbook for Child Health Services [30]. Such guidelines could provide useful knowledge and be a methodological support for the school nurse profession.

Communicating children´s weight to parents is a challenging issue for school nurses. In this study, the school nurses experienced difficulties and concern regarding how to communicate weight deviation to parents and felt uneasiness about how to tackle the subject. This is in accordance with previous research in UK, where health professionals described discussing the issue of children´s overweight and obesity with families and children as sensitive, and strategies for communicating were needed [12]. In this study, the school nurses experienced greater worry, and faster action was taken, when notifying parents that their child was underweight rather than overweight. This result is confirmed by Pesch et al., [31], where paediatricians described increased concern about children with insufficient weight gain and followed them more closely. The word obesity was perceived as charged with negative perceptions and connected with guilt, and the school nurses felt they needed to be careful in their discussions with the parents. This corresponds with another study in Sweden, where the parental reaction determined how the nurses tailored their approach and conversation with parents [32]. School nurses’ barriers to discussing weight issues with parents might result in consequences for public health work to prevent and counteract overweight and, obesity, as well as underweight, in children. The need for further research on the barriers and challenges involved in the conversation with parents concerning overweight and obesity is indicated, as is the understanding of the disparate management regarding overweight contra underweight by the school nurses.

### Strengths and limitations

To ensure the trustworthiness of the study [33], the data was based upon interviews of 16 participant SHS nurses from both municipal and independent school settings. These nurses, worked in elementary schools in both rural and urban context in five different municipalities. The participant school nurses had a wide range of work experience, from 1.5 years to 23 with different supplementary training in their education. This strengthened the transferability of the study. A limitation of the study might be the actual age of the participants was not asked for in the interviews only year of employment, as work experiences might positively affect the communication with children. However, nurses of different age groups/ages might have different attitudes towards communication about weight development and obesity to children and their parents and this might have limited the results. Another limitation to the study could be that all 16 nurses were female, although this reflects the actual context of school nurses in Sweden. The recruitment was accomplished by interested school nurses personally contacting the first author, as well as through snowball sampling, and this could be a potential limitation of the study since it might be that the participant school nurses had an individual interest in questions regarding overweight and obesity in schoolchildren. A broader description of the context of SHS was thus added to strengthen the transferability of the study.

A pilot interview with a school nurse was carried out to test the questions in the semi-structured interview guide prior to the interviews taking place in each school nurses’ undisturbed work office upon the school nurses request, which strengthens the credibility of the study. Each school nurse was asked five semi-structured questions following the interview guide, with supplementary questions asked when needed. This, method might have affected the school nurses in their understanding or influence of giving expected answers and could therefore be a seen as a limitation to the study. To ensure the quality of the data, the interviews were taped and transcribed following the interviews by the first author. The procedures of the content analysis were carried out following a systematic process [22], using tables for documentation and involving all the authors in the research group during the analysis to ensure the credibility of the study. All authors participated continuously in the analysis and creation of categories and subcategories to reach a consensus concerning the result. Examples of the analysis process are integrated into the study, and the categories and subcategories are illustrated with citations to enhance the credibility of the results.

The first author had a preunderstanding of the work as child health nurse. It is essential to acknowledge that the preunderstanding might have influenced the research questions, the interview situation, as well as the interpretation of the study. However, this could in the same manner have deepened the understanding of the context and contributed to strengthening the confirmability of the study. Overall, the findings conform with other studies regarding the communication between health care personnel and parents, as well as strengthen the dependability of the study.

## Conclusion

School nurses are key health professionals in interventions targeting the early onset of overweight and obesity in childhood, and this study indicates the need for training, skills, tools, and strategies to communicate growth data and weight development with parents. A lack of guidelines and knowledge in discussing weight-related topics with parents was described by the school nurses. Furthermore, communicating children´s weight to parents was challenging for school nurses as the topic is charged with weight stigma as well as increased concern by parents. The parents’ right to information concerning their child’s health and growth was perceived as central by the school nurses. They recognised that this information should not stay within SHS but needed to be communicated to the parents. A variety of ways to communicate between school nurses and parents was presented, and this shows an inconsistency in how SHS was offered, as well as a need for the development of consistent and evidence-based procedures for the communication of growth data and weight development to parents.

## Data Availability

The dataset generated and analysed during the current study are not publicly available due to promised confidentiality to study participants but are available from the corresponding author on reasonable request.

## Abbreviations

BMI: Body Mass Index
SHS: School Health Services
